# FINDINGS FROM A PILOT PRAGMATIC CLINICAL TRIAL EMBEDDING CAREGIVER PROGRAMS IN OUTPATIENT SETTINGS

**DOI:** 10.1093/geroni/igad104.0696

**Published:** 2023-12-21

**Authors:** Richard Fortinsky, Kenneth Hepburn, Carolyn Clevenger, Karina Berg, Melinda Higgins, Tori Pascoe, Laura Medders, Vicky Aldrich

**Affiliations:** University of Connecticut, Farmington, Connecticut, United States; Emory University, Atlanta, Georgia, United States; Nell Hodgson Woodruff School of Nursing, Emory University, Atlanta, Georgia, United States; University of Connecticut, Farmington, Connecticut, United States; Emory University, Atlanta, Georgia, United States; University of Connecticut, Farmington, Connecticut, United States; Emory University, Atlanta, Georgia, United States; University of Connecticut, Farmington, Connecticut, United States

## Abstract

Many interventions targeting family/informal caregivers of community-dwelling persons living with dementia have been tested in clinical trials, but few efficacious interventions have been offered in “real world” settings using embedded pragmatic clinical trial (ePCT) methods. This pilot ePCT engaged outpatient geriatrics and memory care clinics in two health systems to implement two caregiver support programs. We used patient portals in electronic health records (EHR) and caregiver registries to invite caregivers to receive one of two remotely-delivered caregiver support programs: the efficacious partly-synchronous Tele-Savvy (TS) program; or the completely-asynchronous Caregiving During Crisis (CDC) program. Random assignment (3TS:2CDC) was employed. Outcome measures were: self-reported caregiving mastery; perceived stress; and caregivers’ reaction to behavioral issues. Measures were collected using self-administered questionnaires sent via patient portals or secure email messaging before randomization and 3 months later (3m). In total, 74 caregivers participated: 59.5% received TS; 73.0% females, 17.6% Black caregivers, with average age 65.5+/-11.8 years. Thirty-four (45.9%) caregivers completed 3m. Compared to the caregivers in CDC, the TS caregivers experienced moderate-to-large effect size improvements in caregiver mastery and frequency of depressive behaviors (mastery: TS Cohen’s d=0.65, CDC d=0.00; depressive frequency: TS d=-0.64, CDC d=-0.04); caregivers in both groups reported moderate decrease in stress (TS d=-0.68; CDC d=-0.54). Implementation evaluation findings included: successful EHR buildout to capture and store caregiver-reported outcomes at one site; recruitment challenges due to caregiver difficulties gaining patient portal access and disinterest in randomization; and sub-optimal provider engagement in inviting caregivers to join the study through the EHR.

